# Variation in outpatient emergency department utilization in Texas Medicaid: a state-level framework for finding “superutilizers”

**DOI:** 10.1186/s12245-017-0157-4

**Published:** 2017-12-04

**Authors:** Chris Delcher, Chengliang Yang, Sanjay Ranka, Joseph Adrian Tyndall, Bruce Vogel, Elizabeth Shenkman

**Affiliations:** 1 Institute for Child Health Policy, Department of Health Outcomes and Policy, University of Florida, Gainesville, FL USA; 2 Department of Computer and Information Science and Engineering, University of Florida, Gainesville, FL USA; 3 Department of Emergency Medicine, University of Florida, Gainesville, FL USA

**Keywords:** Emergency department, Frequent users, Superutilizers, Medicaid

## Abstract

**Background:**

Very frequent outpatient emergency department (ED) use—so called “superutilization”—at the state level is not well-studied. To address this gap, we examined frequent ED utilization in the largest state Medicaid population to date.

**Methods:**

Using Texas Medicaid (the third largest in the USA) claims data, we examined the variability in expenditures, sociodemographics, comorbidities, and persistence across seven levels of ED utilization/year (i.e., 1, 2, 3–4, 5–6, 7–9, 10–14, and ≥ 15 visits). We classified visits into emergent and non-emergent categories using the most recent New York University algorithm.

**Results:**

Thirty-one percent (*n* = 346,651) of Texas Medicaid adult enrollees visited the ED at least once in 2014. Enrollees with ≥ 3 ED visits accounted for 8.5% of all adult patients, 60.4% of the total ED visits, and 62.1% of the total ED expenditures. Extremely frequent ED users (≥ 10 ED visits) represented < 1% of all users but accounted for 15.5% of all ED visits and 17.4% of the total ED costs. The proportions of ED visits classified as non-emergent or emergent, but primary care treatable varied little as ED visits increased. Overall, approximately 13% of ED visits were considered not preventable or avoidable.

**Conclusions:**

The Texas Medicaid population has a substantial burden of chronic disease with only modest increases in substance use and mental health diagnoses as annual visits increase. Understanding the characteristics that lead to frequent ED use is vital to developing strategies and Medicaid policy to reduce high utilization.

## Background

According to the National Center for Health Statistics, 6.9% of adults aged 18 and over had two or more emergency department (ED) visits in the past 12 months (2013) [1]. The proportion of Medicaid beneficiaries utilizing the ED to this extent is more than twice that of non-Medicaid populations. That is, 19% of Medicaid recipients had two or more visits compared to 3.9 and 8.1% of those privately insured or with no coverage [1]. The disproportionate representation by Medicaid recipients appears to persist at the highest levels of ED utilization. One systematic review found that publicly insured populations were over-represented among “frequent” ED users [2]. At the state level, in South Carolina, Chen et al. found that Medicaid recipients showed more frequent and avoidable ED use [3]. National estimates of the cost of ED care vary from 2 to 12.5% of total health care expenditures totaling approximately $328 billion in 2010 [4, 5]. Medicaid’s proportion of these costs ranges from $27 billion to $47 billion annually [6]. More specifically, an estimated $64.4 billion is spent on potentially avoidable ED encounters [5]. National estimates of Medicaid’s proportion of the cost of avoidable visits are not available, but they appear to be substantial if consistent with state-level studies. In a study in Washington State, approximately 12% of visits that Medicaid enrollees made could have been avoided. Using a different methodology, the state of Minnesota estimated that two thirds (67%) of ED visits were potentially preventable and Medicaid enrollees accounted for approximately 41% of these. Given these findings, interest in identifying beneficiaries at the highest ends of the ED utilization distribution, sometimes referred to as Medicaid “superutilizers,” has emerged [7].

Medicaid plays an important role in caring for high-need, high-cost (HNHC) patients, heightening the importance of identifying strategies and interventions to control costs while providing needed services to beneficiaries [8, 9]. In their review, LaCalle and Rabin found that frequent users comprise 4.5 to 8% of the ED-utilizing population and 21 to 28% of all visits [2]. In one of the largest studies to date, Billings and Raven reported that, among Medicaid enrollees visiting EDs in New York City in 2007, 10.3% visited five or more times, representing 34.2% of all ED visits [7]. These utilization patterns have been attributed to multiple factors such as being white and insured, behavioral health status and substance abuse, chronic disease burden, access to a usual source of care, deficiencies in quality and/or continuity of care, ED referral practices, limited primary care availability, and social determinants of health [2, 7, 10–13]. State-level, population-based efforts to address the health needs of these high utilizers start with a robust characterization of these beneficiaries from statewide data sources [12, 13]. In Maryland, Horrocks et al. reported that, in the absence of state data, analyses limited to a single hospital would fail to identify two out of five high utilizers (≥ 5 ED visits) [14].

Little research has been published using statewide data. The work of Billings and Raven [7] is the largest examination of high-utilizing Medicaid beneficiaries to date, but the study is limited in geographic scope to New York City (NYC) and may not represent the experience of other large, diverse state Medicaid programs. The purposes of this paper are to (1) examine the characteristics of ED utilizers in the State of Texas’ Medicaid population which, as of May 2016, is the third largest in the USA and (2) compare the Texas experience with the NYC experience documented by Billings and Raven [15].

## Methods

### Approach

For this analysis, we replicated and extended the analytic framework of Billings and Raven [7], using 2014 administrative data available from the Texas Medicaid program. The objective was to examine the variability in health care expenditures, demographic characteristics, health conditions, and comorbidities across seven levels of ED utilization (i.e., 1, 2, 3–4, 5–6, 7–9, 10–14, and 15 or more outpatient visits). The University of Florida Institutional Review Board approved this study and granted a full waiver of informed consent (IRB201401068).

### Study population

Texas has the third largest Medicaid enrollment (*n* = 4.7 million) in the USA, representing approximately 7% (2014) of the US Medicaid population [15–17]. The Texas Health and Human Services Commission (HHSC) administers Medicaid using both a managed care model consisting of twenty-seven (27) managed care organizations (MCOs) and a fee-for-service (FFS) program. Managed care is delivered in three programs—the State of Texas Access Reform (STAR) program, State of Texas Access Reform Plus (STAR+PLUS), and STAR Health—each serving distinct clinical populations. For this analysis, the programs were divided into two categories: managed care and FFS. Medicare claims were not available for this analysis; therefore, individuals dually enrolled in Medicare and Medicaid were excluded.

### Operational definitions

#### Emergency department visits

An emergency department (ED) visit was defined by a facility claim with a revenue code of “045x.” If revenue codes were not available for the facility claim, Current Procedural Terminology (CPT) codes “99281” through “99285” were used to identify ED visits. Visits resulting in an inpatient admission within 48 h (current and following day) for the same or similar primary diagnosis were not considered ED visits. Visits, as opposed to enrollees, were attributed to a managed care or FFS program regardless of whether the enrollee changed programs during the study period. ED visits were categorized using the Billings and Raven analytic categories (i.e., 1, 2, 3–4, 5–6, 7–9, 10–14, and 15 or more visits) [7]. Given that there is no scientific consensus on what constitutes “overutilization” based on visit frequency, we defined a range of *potential* overutilization starting at three or more visits and extending to 10 or more visits which we considered “extremely frequent” users [18].

#### Acute hospital inpatient admissions

An acute inpatient (IP) admission (i.e., a hospital stay) was identified by a billing type with the prefix “11x.”

#### Medical expenditures

For ED and IP setting-specific expenditure calculations, only institutional claims were used. For total medical expenditure calculations, institutional and professional claims were included. Pharmacy claims were excluded. The mean expenditure per unique enrollee was calculated by dividing the sum of the paid amount by Medicaid for each enrollee in the subgroup by the number of enrollees. The mean ED and IP expenditure per visit or stay was calculated by dividing the sum of the paid amount of Medicaid for each enrollee in the subgroup by the number of ED visits or IP stays in the subgroup. The percent of total medical expenditures were calculated by dividing the sum of the total paid amount of Medicaid across all subgroups by the sum of the total paid amount by Medicaid for enrollees in the specific subgroup.

#### Enrollee sociodemographics

Enrollee-level information included demographic variables including age at enrollment, sex, race/ethnicity (black, Hispanic, white, and other/unknown), residential address, and residential county. Age, residency, and program eligibility were determined as of 2014. The age range was limited to 18–62 years [7]. Physical addresses were geocoded to the census tract level to determine the enrollees’ neighborhood poverty context defined as the percent of the census tract population living under 100% of the federal poverty line (2010) [19]. A neighborhood was considered high poverty if 20% of the households lived below federal poverty [20].

Geocoding was performed with ArcGIS 10.3.1 using ESRI Premium Streets Data 2014. Ninety-two percent of all physical addresses were geocoded to the census tract level. Enrollees with missing addresses were included in the overall analysis, but the poverty measure was calculated from enrollees with available geographic data. An enrollee’s county of residence was classified as low density if the population density was less than 100 inhabitants per square mile.

#### Diagnostic history

Patients’ diagnostic history was based on *International Classification of Diseases*, Ninth Revision, Clinical Modification (ICD-9-CM) codes from all 25 available diagnosis fields from all available claims unless otherwise indicated. A history of medical conditions was determined by examining data from 2011 to 2014 using ICD-9-CM codes provided by Billings and Raven. The number of ED providers was calculated as the number of unique national provider identifier (NPI) codes associated with the ED visit. Some NPIs may not be correctly attributed to facilities if billing is handled through third-party organizations.

The weighted Charlson Comorbidity Index was used to describe the overall burden of disease and takes into account the number and the seriousness of comorbid diseases. The weighted index (ranging from 0 to 33) was used in this analysis [21].

#### New York University ED profiling algorithm

The New York University (NYU) ED profiling algorithm was used to classify ED visits for enrollees whose visits were not due to injury or behavior health issues [22]. The updated version with ICD-10 codes was used. The algorithm delineates ED use into “emergent” and “non-emergent.” “Non-emergent” was considered “primary care treatable.” “Emergent” was sub-divided into “ED care needed” and “primary care treatable.” Lastly, “ED care needed” was divided into “not preventable or avoidable” and “preventable or avoidable.”

#### Frequent and persistent users

For longitudinal analyses, frequent users were classified into four groups based on the total number of ED visits and the number of follow-up years meeting the pre-determined visit counts. The latter is a measure of persistence. The four groups were enrollees with (1) three or more ED visits in the index year and three or more ED visits in the following year, (2) five or more ED visits in the index year and five or more ED visits in the following year, (3) three or more ED visits in the index year and three or more ED visits in the two subsequent years, and (4) five or more ED visits in the index year and five or more ED visits in the two subsequent years.

#### Annualized visits

To standardize ED visit counts when beneficiaries were not enrolled continuously throughout the calendar year, the counts for each year were annualized. The annualized counts are calculated as the actual counts of ED visits made by a beneficiary divided by the proportion of the year for which the beneficiary is enrolled in Medicaid.

### Statistical analyses

Cross tabulations of medical, sociodemographic, and health conditions are presented by categories of ED utilization for calendar year (CY) 2014. Cross tabulations using CY 2012 as the index year and 2 years of follow-up (the most recent data available) are also presented for annualized ED visits, emergent versus non-emergent conditions, and persistence by categories of ED utilization.

## Results

### Characteristics of ED users

**Fig. 1:**
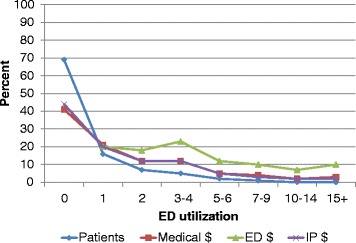
Percent of total patients and medical, emergency department, and inpatient dollars by ED utilization category, Texas Medicaid enrollees, 2014

Table 1 shows the profile of medical expenditures, sociodemographic, and health-related conditions of adult Texas Medicaid enrollees in CY 2014. Thirty-one percent (*n* = 346,651) of Texas Medicaid adult enrollees visited the ED at least once in 2014. Patients in the range of potential overutilization (three or more ED visits) accounted for 8.5% of all adult patients, 60.4% of the total ED visits, 26.4% of the total medical costs, and 62.1% of the total ED expenditures. Extremely frequent ED users (10 or more ED visits) represented less than 1% (0.72%) of all ED users but accounted for 15.5% of all ED visits, 17.4% of the total ED costs, and 5.0% of the total medical expenditures. Figure 1 shows the percent of total patients and medical, ED, and IP expenditures by ED utilization category. Mean ED expenditures per patient for patients with 15 or more ED visits was 2.3 times the mean for patients with 10–14, $10,750 versus $4722 respectively.

**Table 1:** Characteristics of adult emergency department (ED) users in Texas Medicaid, CY 2014

	Number of ED visits								
				Range of potential overutilization					
								EF^a^	
	0	1	2	3–4	5–6	7–9	10–14	15+	All
Number of patients	773,001	176,198	75,955	57,435	19,132	9970	4877	3084	1,119,652
Percent of patients	69.04	15.74	6.78	5.13	1.71	0.89	0.44	0.28	100.00
Percent of ED visits	0.00	21.25	18.32	23.23	12.42	9.32	6.75	8.71	100.00
Cumulative percentage of ED visits	0.00	50.83	72.74	89.31	94.83	97.70	99.11	100.00	NA
Medical expenditure^b^									
Percent of total medical expenditure	40.72	20.58	12.28	12.31	5.48	3.66	2.39	2.57	100.00
Average medical expenditure per patient	$2145	$4758	$6582	$8731	$11,673	$14,945	$19,968	$33,989	$3637
ED expenditure^c^									
Percent of total ED expenditure	NA	20.38	17.56	22.83	12.29	9.56	7.12	10.25	100.00
Average ED expenditure per patient	NA	$374	$747	$1285	$2076	$3101	$4722	$10,750	$289
Average ED expenditure per visit	NA	$369	$368	$377	$379	$392	$400	$433	$382
Inpatient (IP) stays									
Average IP stays	0.23	0.37	0.45	0.57	0.77	0.97	1.38	2.35	0.31
Percent of total IP stays	51.04	18.53	9.91	9.47	4.23	2.79	1.94	2.08	100.00
Percent of total IP expenditure^c^	43.74	19.80	11.61	11.61	5.13	3.50	2.31	2.30	100.00
Average IP expenditure per patient^c^	$830	$1649	$2243	$2967	$3932	$5159	$6938	$10,969	$1311
Average IP expenditure per stay^c^	$3560	$4413	$4820	$5035	$4859	$5102	$4847	$4553	$4130
Program visit distribution^d^ (%)									
Fee-for-service (FFS)	NA	32.75	28.58	24.23	20.94	19.19	18.15	19.08	25.07
In managed care	NA	67.25	71.42	75.77	79.06	80.81	81.85	80.92	74.94
Sociodemographic characteristics									
Mean age (years)^e^	30.89	32.64	33.14	33.79	34.73	35.88	37.28	38.94	31.63
Percent female	78.18	77.82	79.21	79.67	79.10	77.60	74.98	67.48	78.23
Race or ethnicity (%)									
Black	18.68	21.39	23.67	25.26	26.4	26.25	24.48	23.44	20.02
Hispanic	46.26	41.75	37.65	33.82	29.19	27.40	24.50	25.23	43.72
White	23.55	25.63	27.15	28.5	30.27	31.18	32.93	32.3	24.62
Other or unknown	11.51	11.22	11.53	12.41	14.15	15.16	18.09	19.03	11.64
Poverty index^f^	22.86	23.50	23.53	23.44	23.26	23.78	23.53	24.25	23.06
High-density population	81.76	80.76	80.80	80.74	81.36	81.52	82.20	82.60	81.48
Disability status (%)									
Disabled eligibility	23.04	32.20	36.12	40.82	47.67	54.62	62.91	75.45	27.30
History of chronic conditions^g^									
Any chronic condition (%)	26.42	45.42	53.49	62.17	71.98	79.31	86.45	93.87	34.78
Multiple chronic conditions (%)	13.48	26.07	32.5	39.83	49.72	58.95	69.57	83.53	19.56
Substance use disorders^h^ (%)	13.85	31.82	41.57	50.72	61.37	69.25	78.55	85.41	22.23
Mental illness^h^ (%)	22.88	39.83	49.12	58.54	70.20	78.15	86.22	90.08	30.92
Schizophrenia^h^ (%)	2.98	5.38	7.03	9.23	13.32	16.99	22.06	29.31	4.41
Bipolar disorder^h^ (%)	4.76	10.45	14.44	19.20	27.40	33.85	40.78	49.29	7.98
Depressive psychosis^h^ (%)	5.33	10.81	14.43	18.54	25.03	30.18	36.19	46.24	8.29
Number of chronic conditions	0.66	1.14	1.42	1.77	2.23	2.66	3.25	4.27	0.93
Charlson Comorbidity Index^i^	0.63	1.24	1.55	1.93	2.42	2.90	3.57	4.94	0.93

Authors’ analysis of Texas Medicaid claims, encounter, and enrollment data. This analysis excludes dual-eligible enrollees

*NA* not applicable

^a^Extremely frequent (EF) ED users

^b^Includes professional and institutional expenditures. Excludes pharmacy expenditures

^c^Includes institutional expenditures

^d^The percentage of managed care calculated is based on enrollment (e.g. if a patient has two ED visits and one occurs while enrolled in FFS and the other in managed care, one visit is counted as FFS and the other as managed care)

^e^Age inclusion 18–62 years old

^f^The average percentage of people living in poverty in the enrollees’ census tract (2010 US census data)

^g^The history conditions (Chronic conditions, substance use disorder, mental illness, schizophrenia, bipolar disorders, depressive psychosis, and the Charlson Comorbidity Index are identified from 2011 to 2014 diagnosis codes

^h^The ICD-9-CM codes used to define chronic conditions, SUD, mental illness, schizophrenia, bipolar disorder, and depressive psychosis were provided by Billings and Raven [7]

^i^The weighted version of the Charlson Comorbidity Index is used here. The range of index is 0–33

Patients with 15 or more ED visits, on average, had six times the number of acute inpatient (IP) stays compared to users with only one ED visit (2.4 vs. 0.4 stays, respectively). The extremely frequent ED users accounted for approximately 4% of the total IP stays and costs and had the highest mean IP expenditures per patient ($6939 and $10,969). Mean IP expenditures per patient for patients with 15 or more visits were 1.6 times the mean for patients with 10–14 visits, $10,969 versus $6938 respectively. The majority of patients were enrolled in managed care, with greater than 75% of those with three or more visits in managed care. The percent of enrollees that lived in a high-poverty (> 20% of households) neighborhood varied little.

Most sociodemographic characteristics only varied by a couple of percentage points across the entirety of the ED utilization spectrum with the exception of female sex and Hispanic race/ethnicity. Extremely high-frequency ED patients, those with 15 or more visits and 10 to 14 visits, were 67 and 75% female, respectively. The percent of Hispanic patients declined from 42% with one ED visit to 25% in the 15 or more visit category. Considerable variability in this range was also found for having any chronic condition (2.1 times), multiple chronic conditions (3.2 times), substance use disorders (SUDs) (2.7 times), and mental illness (2.3 times). The prevalence of each of these conditions was greater than 80% for patients with 15 or more ED visits. The diagnosis of specific mental health conditions—schizophrenia, bipolar disorder, and depressive psychosis—resulted in higher observed variability in this range than from the more general “mental illness” category. Specific diagnosing increased the observed prevalence of these conditions by 5.4, 4.7, and 4.3 times, respectively. Approximately one third to half of the extremely frequent ED users had a history of these conditions. As expected, disease severity, as measured by both the number of chronic conditions and the Carlson Comorbidity Index, increased across the range.

Table 2 shows ED visit-level characteristics in 2012 and the subsequent 2-year follow-up period (2013, 2014). For example, if all beneficiaries were enrolled continuously, the estimated accrual is estimated at 3.9 ED visits in 2012. The annualized count decreases by more than half, to 1.72 and 1.35 ED visits, in subsequent years reflecting a lower average intensity of use through time. As expected, the extremely frequent ED users had the highest annualized counts (27.5) of all utilization categories; their intensity of ED use also decreases in subsequent years but remains relatively high (16.4 and 11.4). In other words, when an enrollee visited the ED 15 or more times in 2012, 2 years later, the average utilization was still equivalent of an enrollee with 10 to 14 ED visits.

**Table 2:** Adult emergency department (ED) visits in index (2012) and subsequent years (2013, 2014) in Texas Medicaid

	Number of ED visits in index year (2012)^a^							
			Range of potential overutilization					
							EF^b^	
	1	2	3–4	5–6	7–9	10–14	15+	All
Annualized ED visits, index year	2.43	3.71	5.03	7.07	9.69	13.78	27.47	3.94
Annualized ED visits, 1 year after	0.91	1.42	2.21	3.49	5.28	8.03	16.36	1.72
Annualized ED visits, 2 years after	0.74	1.15	1.73	2.78	4.00	5.94	11.46	1.35
Number of ED providers, index year^c^	1.00	1.34	1.65	2.01	2.39	2.88	4.26	1.32
Primary diagnosis, visits in index year^d^ (%)								
Chronic condition	3.79	4.04	4.39	5.05	5.76	6.49	9.73	5.02
Substance use disorder	0.62	0.61	0.71	0.76	1.02	1.14	1.33	0.79
Mental illness	2.00	2.12	2.37	2.66	3.16	3.55	4.15	2.59
Diagnoses 1–3, visits in index year^e^ (%)								
Substance use disorder	7.03	7.43	8.17	8.97	9.82	10.47	10.73	8.41
Mental illness	5.33	5.93	6.87	7.99	9.25	10.71	11.74	7.40
NYU algorithm preventable events^f^ (%)								
Injury	14.07	13.02	12.60	12.51	12.92	13.04	12.16	13.01
Non-emergent	24.92	25.28	26.00	26.00	25.29	25.09	22.21	25.16
Emergent, primary care treatable	22.53	23.04	22.79	22.64	22.38	22.18	23.14	22.72
Emergent, preventable, or avoidable	5.12	5.34	5.62	6.02	6.21	6.12	5.94	5.62
Emergent, not preventable, or avoidable	12.66	12.63	12.18	12.01	12.13	12.65	15.53	12.67
Frequent users, index year and 2 years after (%)								
3+ visits each year	NA	NA	10.98	21.68	34.07	47.04	60.24	5.01
5+ visits each year	NA	NA	NA	9.29	19.68	32.42	50.47	1.91
Enrolled in Medicaid in the subsequent 2 years	51.00	57.34	63.03	68.89	72.25	75.99	76.05	56.42

Authors’ analysis of Texas Medicaid claims, encounter, and enrollment data. This analysis excludes dual-eligible enrollees

*NA* not applicable

^a^The index year is set to 2012 to provide a 2-year prospective window

^b^Extremely frequent ED users

^c^Calculated using the total number of unique national provide identifier (NPI) numbers; unique NPIs, however, may be attributed to facilities or physicians

^d^The ICD-9-CM codes used to define chronic condition, SUD, and mental illness were provided by Billings and Raven [7]

^e^Includes primary, secondary, and tertiary diagnoses

^f^The NYU ED profiling algorithm is used here

The percentage of ED visits where the principal diagnosis was a chronic condition ranged from 4.0 to 9.7% in the range of potential overutilization. The prevalence of SUDs and mental illness was less than 5% across the spectrum. As expected, the prevalence increased and ranged from 6.9 to 11.7% when the first three diagnostic codes were used in the analysis.

The percentage of ED visits classified as non-emergent or emergent, but primary care treatable varied little as the number of ED visits increased. Overall, approximately 13% of ED visits were considered not preventable or avoidable using the NYU algorithm in this Medicaid population. This percentage only increased to 15.7% for extremely frequent users. This finding is consistent with the Billings and Raven [7] argument that the degree to which these visits are avoidable does not appear to change dramatically with utilization frequency.

High utilizers were more likely to be continuous ED users. Approximately 60.2% of the users in the 15 and more visit category in the index year had three or more visits in the following 2 years and 50.4% had five or more visits.

### Limitations

This work has several limitations. First, the analysis is largely descriptive. Additional statistical analyses are needed to test specific hypotheses. Second, while Texas is a very large, diverse state, it is not clear to what extent our results generalize to the overall Medicaid population nationally. Third, while the validity of the NYU algorithm was found acceptable in commercial, Medicare, and the general population, the algorithm has not undergone the same direct testing for Medicaid populations [23, 24]. Chen et al. found acceptable correlation between the algorithm and several measures of severity in the South Carolina ED population which included Medicaid recipients [3].

## Discussion

In this study of emergency department use in Texas Medicaid, we examined the variation in key dimensions associated with healthcare utilization including high-frequency use, sociodemographics, setting, cost concentration, chronic/comorbid conditions including mental illness and SUDs, inappropriate or avoidable visits, and persistence. We briefly discuss the key findings.

### Sociodemographics

Overall, females and Hispanics are the predominant users of ED services in the Texas Medicaid population. This finding is consistent with a nationally representative sample that found that Hispanic/Latino ethnicity was associated with higher ED utilization [25]. However, representation for both groups declined appreciably at the higher levels of utilization. In 2014, enrollees generally lived in high-density counties (i.e., more urban) and in neighborhoods where one quarter of the households had incomes below federal poverty guidelines. We note that the geocoding rate to the census tract level was high for this population and depends heavily on acquiring a proper street address. Follow-up analyses, however, indicated that address information may be differentially missing for the highest ED-utilizing enrollees. Future work may investigate the extent to which address information may be a marker for social vulnerability in this population.

### Setting-specific, high-frequency use

Extremely frequent users (10 or more ED visits) represented 2.3% of the ED using population and accounted for 15.5% of all ED visits. Unlike other studies, our analysis also examined the inpatient stay profile in this outpatient ED population. The extent to which the ED high utilizers would be considered inpatient high utilizers is, of course, dependent upon which definition is used. For example, the Agency for Healthcare Research and Quality defines superutilization as ≥ 4 inpatient stays per year which is nearly two times higher than the mean number of inpatient stays accumulated by the extremely frequent users in our study [26]. Alternatively, [27] defined inpatient superutilization as ≥ 3 inpatient stays per year or ≥ 2 stays with a concurrent mental health diagnosis per year. When we applied these definitions, we found that 24 and 40%, respectively, of extremely frequent ED users would also be considered IP superutilizers. We note that our study was focused on outpatient ED utilization, so enrollees that were admitted to the hospital via the ED were excluded.

### Cost concentrations

Our results show that extremely frequent ED users represent less than 1% of enrollees but account for 5.0% of total medical expenditures, 4.6% of the total IP expenditures, and 17.4% of total ED expenditures. While extremely frequent ED users account for relatively small percentages of health care expenditures, such high ED use may reflect a lack of access to primary care or inadequate quality of such care.

### Chronic, comorbid conditions, mental illness, and SUDs

It is in this domain that our findings show the largest divergence from that of Billings and Raven [7]. In Texas, the number of chronic conditions diagnosed in Texas enrollees in the highest utilization levels is approximately two times higher than in the NYC population. Simply put, there is a more substantial chronic disease load in Texas than NYC among these high utilizers. Second, enrollees in the Texas Medicaid population appear to *principally* present far less often for SUDs and mental illness. For example, the percentage of ED visits associated with SUDs at the highest end of the utilization spectrum (i.e., 15 or more visits) in NYC was approximately 15 times higher than that of Texas. Even with a more conservative approach using secondary diagnosis codes, the observed prevalence is still approximately 2.5 times higher in NYC. Third, a substantial increase in the risk of SUDs between the 10 to 14 category and 15 or more category is striking in NYC (10 to 24%, respectively). This very sharp increase in SUD risk among extremely high utilizers is absent in the Texas Medicaid population.

It is not clear if these state-level differences are due to underlying variation in medical coding practice, time period, population rates for SUDs and/or utilization of mental health services, or other factors. Hispanics represent more than half (54%) of Medicaid enrollees in Texas compared to 28 and 25% for New York and the USA, respectively [28]. SUDs and mental illness may be underestimated due to racial and ethnic disparities that decrease the likelihood of diagnosis and treatment of SUDs and mental health disorders in these populations [29–31]. An analysis by Rinehart et al. identified a subset of high utilizers that were predominantly Hispanic with complex medical conditions but fewer behavioral health problems [32]. Overall, estimates of SUD prevalence from the National Survey on Drug Use and Health (2010) indicate that 10.1% of the Texas Medicaid population had a SUD compared to 13.4% of this cohort in New York State [33, 34]. We note that our SUD estimates using claims data are consistent with these survey-based estimates. In summary, if the conventional wisdom about high utilizers is that SUDs are highly prevalent and increasing sharply as a function of utilization, it is not observed in the Texas Medicaid population. The reasons for this finding are not clear and warrant further exploration.

### Inappropriate and/or avoidable visits

Medicaid enrollment has been associated with higher rates of potentially avoidable ED visits, and this proportion varies from state-to-state [35]. Approximately one quarter of the ED visits in the Texas Medicaid program were not considered emergencies. By comparison, using the same NYU algorithm, Mississippi reported that more than half of the ED visits in that state were non-emergent [36]. As noted in other populations, the percentage of ICD-9-CM-based conditions considered preventable or avoidable varied little as utilization increased except for the most extreme utilizers. Even in this group, the percentage of avoidable visits increased modestly. Given the limitations of the NYU algorithm, future research should compare alternative measures of potentially preventable conditions [3].

### Persistence

Persistence of utilization, as measured by whether an enrollee had five or more visits in two subsequent years, was stable. Among the extremely high utilizers, approximately one third to half of the enrollees visited EDs at this rate (or higher) for 3 years in a row.

## Conclusions

This is one of the only statewide studies of Medicaid emergency department use to date and as such adds findings from a large, diverse state to the literature. By adopting the published Billings framework for the analysis, the findings can be replicated in other states. Furthermore, we extended the analysis to include inpatient expenditure information for outpatient ED high utilizers and measures of social determinants of health in this population. Also, we provide a 3-year longitudinal perspective of persistency providing the basis for analysis of change and predictive analytics moving forward.

Our Texas Medicaid results tend to confirm the findings of Billings and Raven in their New York City sample. We find that the ED-utilizing Texas Medicaid population has a substantial burden of chronic disease. Similarly, we not only find relatively modest increases in substance use and mental health diagnoses as ED use increases; our results lack the dramatic surge in substance use diagnoses in the highest ED visit categories found in New York City. Non-emergent ED use in Texas remains fairly constant across ED use categories and falls in the same 20–30% range found by Billings and Raven. Finally, the disproportionate inpatient use and expenditures found among high ED use categories in Texas are consistent with high burdens of disease that extend beyond simply high ED use. Indeed, there is substantial overlap between the ED and inpatient populations at the highest ends of the utilization spectrum.

“Superutilizers” are a complex population with substantial medical problems that need to be addressed in a careful and coordinated fashion. Future work on this population will need to be informed by these characteristics and recognize that reducing high utilization may not be as easy or straightforward as originally hoped.

## Abbreviations

CPT: Current Procedural Terminology
CY: Calendar year
ED: Emergency department
FFS: Fee-for-service
HHSC: Health and Human Services Commission
HNHC: High-need, high-cost
ICD-9-CM: International Classification of Diseases, Ninth Revision, Clinical Modification
IP: Inpatient
MCO: Managed care organization
NPI: National provider identifier
NYC: New York City
STAR+PLUS: State of Texas Access Reform Plus
STAR: State of Texas Access Reform
SUD: Substance use disorder
